# Do the effects of krill oil supplementation on skeletal muscle function and size in older adults differ by sex, age or BMI: A secondary analysis of a randomised controlled trial

**DOI:** 10.1016/j.jnha.2025.100747

**Published:** 2025-12-03

**Authors:** Oliver Hayman, Saleh AA Alkhedhairi, Emile Combet, Terry J Quinn, Angus M Hunter, Stuart Goodall, Stuart R. Gray

**Affiliations:** a School of Cardiovascular and Metabolic Health, College of Medical, Veterinary and Life Sciences, University of Glasgow, Glasgow, United Kingdom; b Monash Exercise Neuroplasticity Research Unit, Department of Physiotherapy, School of Primary and Allied Health Care, Faculty of Medicine, Nursing and Health Science, Monash University, Melbourne, Australia; c Department of Medical Biosciences, College of Veterinary Medicine, Qassim University, Saudi Arabia; d School of Medicine, Dentistry and Nursing, College of Medical, Veterinary and Life Sciences, University of Glasgow, Glasgow, United Kingdom; e Department of Sport Science, Nottingham Trent University, Nottingham, United Kingdom; f School of Sport, Exercise, & Rehabilitation, Faculty of Health and Life Sciences, Northumbria University, Newcastle upon Tyne, United Kingdom; g Institute of Sports Science and Innovation, Lithuanian Sports University, Lithuania

**Keywords:** Sarcopenia, Omega-3, Ageing, Sex differences, Strength

## Abstract

This study examined whether the effects of krill oil supplementation on muscle function and size differ by sex, age or BMI in healthy older adults. This was a secondary exploratory analysis of a previous randomised controlled trial. Men and women aged ≥65 years, with BMI < 35 kg/m² and engaging in <1 h per week of structured exercise, were enrolled in a randomised, double-blind, controlled trial (NCT04048096) between March 2018 and March 2020. Participants received either 4 g/day krill oil or a control oil for 6 months. Ninety-four participants were included (Control n = 45; 27 women, 18 men; Krill n = 49; 26 women, 23 men) with muscle size, strength and neuromuscular function measured before and after the intervention period. Responses to intervention were compared between subgroups by sex (male/female), age (≤70 years/>70 years) and BMI (≤24.9 kg/m^2^/>25 kg/m^2^). Increases in muscle strength, size, and physical function in response to krill oil supplementation were comparable across age, sex and BMI subgroups (all P > 0.05). This was similar for neuromuscular measures although increases in the Mwave the response to krill oil supplementation differed by sex, with no change over time in females in either krill or control groups, but an increase in Mwave in males in the krill group (+3.80 [1.72–5.88] mV, p = 0.016) with a tendency for a decrease in the control group (−3.71 [1.58–6.05] mV, p = 0.059). In conclusion, krill oil supplementation improved muscle strength and size in older adults regardless of age, sex and BMI status, although neuromuscular effects of krill oil on membrane excitability, via the Mwave, may be more pronounced in men.

ClinicalTrials.gov Identifier: NCT04048096

## 1 Introduction

The age-related loss of muscle mass and strength, known as sarcopenia, is associated with reduced quality of life, greater risk of falls, and increased healthcare costs, estimated at £2.5 billion annually in the UK [1,2]. The prevalence of sarcopenia is unclear, estimated between 10 and 27% in adults 60 years of age and older [1]. With the percentage of older people (> 65 years) predicted to rise from 19% (12.7 million) in 2022 to 27% (22.1 million) in 2072 in the UK, it is crucial to develop therapies to increase, or attenaute the decline in, muscle strength and mass in older adults. Resistance exercise is effective, even in nonagenarian women [2], but attenuated responses are observed in comparison to young people, due to the so-called “anabolic resistance” [3] along with participation rates being low [4]. Alternative strategies are, therefore, needed.

Building on epidemiological [5,6], cell culture, and animal data [7,8], human research has demonstrated that 8 weeks of long-chain n-3 polyunsaturated fatty acid (LCn-3 PUFA) supplementation (fish oil, 4 g/day) increased muscle protein synthesis (MPS) during a hyperaminoacidaemic-hyperinsulinaemic clamp in older adults [9]. A longer-term study over 6 months indicated that LCn-3 PUFA supplementation (fish oil, 4 g/day) resulted in a 3.6% increase in muscle volume and a 2.3 kg increase in grip strength, in older adults [10]. Similar results have been demonstrated in a larger study with krill oil as the source of LCn-3 PUFA, which also indicated that the effects on muscle strength may be neuromuscular in origin, with an increase in the Mwave, a measure of muscle membrane excitability, observed following LCn-3 PUFA supplementation [11]. Whether these responses differ by key participant characteristics, such as age, sex and BMI, has not been investigated.

When combined with resistance exercise, apparent sex differences have been noted, with LCn-3 PUFA supplementation increasing muscle strength in women (∼34% vs 16% in placebo) but not in men [12]. Early epidemiological evidence also suggests sex-specific associations in the absence of exercise, with each additional weekly portion of fatty fish associated with a 1.8% higher grip strength in women and only a 1.0% higher grip strength in men [5]. There are also some evidence that metabolic/inflammatory responses to LCn-3 PUFA can differ dependent on BMI status [13], but no work has investigated downstream effects on muscle. Furthermore, muscle decline in older age is known to differ by both age and sex [14], but how this influences response to LCn-3 PUFA supplementation remains to be established. Currently, therefore, no randomised controlled trial has examined whether LCn-3 PUFA supplementation alone has sex/age/BMI-specific effects on muscle function, size or neuromuscular function in older adults. Therefore, the present exploratory analysis aimed to determine whether responses to krill oil supplementation differ by sex/age/BMI status.

## 2 Materials and methods

### 2.1 Study design

The current study is an exploratory secondary analysis from a previously published randomised controlled trial [11] (NCT04048096). This was a double blind randomised controlled trial with participants assigned, following baseline assessment, to either control or krill oil groups for the 6-month intervention period. As this analysis was not powered to detect subgroup interaction effects, all sex, age and BMI based comparisons should be interpreted as exploratory

### 2.2 Participants

Men and women aged ≥65 years with BMI < 35 kg/m^2^ and engaging in <1 h per week of structured exercise (defined as planned, repetitive, intentional movement) were recruited from the Glasgow area between March 2018 and March 2020. Recruitment was via posters and newspaper/magazine advertisements. Exclusion criteria included: diabetes mellitus; severe cardiovascular disease; seizure disorders; uncontrolled hypertension (>150/90 mmHg at baseline); active cancer or remission <5 years; ambulatory impairment preventing functional assessments; dementia; medications known to affect muscle (e.g., corticosteroids); implanted electronic devices (e.g., pacemaker, defibrillator, insulin pump); anticoagulant therapy; use of nutritional supplements; seafood allergy; or regular consumption of >2 portions of oily fish per week. The study was approved by the University of Glasgow Medical, Veterinary, and Life Sciences College Research Ethics Committee [Reference 200170067]. It was carried out per the ethical standards in the 1964 Declaration of Helsinki. Written informed consent was obtained after explaining the aims, risks, and potential discomfort associated with the study.

### 2.3 Interventions

The control group received 4 g/day mixed vegetable oil (olive oil [extra virgin, cold pressed], maise oil [refined], palm kernel oil [refined], and medium-chain triglycerides in a 4:4:3:2 ratio). The krill oil group received 4 g/day krill oil (SuperbaBoost™; Aker Biomarine Antarctic AS, Lysaker, Norway). Each 1 g krill oil capsule contained 322 mg LCn-3 PUFA, including 193 mg eicosapentaenoic acid (EPA), 96 mg docosahexaenoic acid (DHA), and 79 mg choline. Capsules were identical in appearance and taste. The manufacturer had no role in study design, conduct, or analysis.

### 2.4 Outcomes

Measurements were performed at baseline and 6 months post supplementation unless otherwise stated.

#### 2.4.1 Knee extensor muscle strength

Muscle strength of the right knee extensors was assessed during an isometric maximal voluntary contraction (MVC) measured using a calibrated dynamometer (Biometrics Ltd, Newport, UK) with the knee angle fixed at 90 °. Participants performed a minimum of three contractions (≤10 s each) with 3 min rest between trials, and the highest value was used for analysis.

#### 2.4.2 Grip strength

Grip strength was assessed using a Jamar dynamometer (J.A. Preston Corporation, USA). Participants were seated with the arm supported and the elbow flexed at 90 °. Three trials were performed with each hand, and the highest value was used for analysis.

#### 2.4.3 Muscle thickness

Muscle thickness of the right *vastus lateralis* muscle was measured using an ultrasound imaging device, as detailed previously [15].

#### 2.4.4 Physical function

Chair-stands were performed with participants rising from a chair with their arms across their chest 5 times; this was repeated 3 times and the quickest time is used for analysis. Following this, three separate 4-m walks were performed, with the fastest time recorded for analysis.

#### 2.4.5 Neuromuscular function

During the measurement of knee extensor muscle strength, a surface EMG (sEMG) electrode was positioned on the vastus lateralis muscle. Following the contraction, sEMG signals were processed root mean square (RMS), and average RMS calculated over a 500 ms period, 250 ms each side of peak force, with rate of torque development also calculated. With participants seated in the dynamometer, an electrode was placed on the vastus lateralis muscle and attached to a constant current variable voltage stimulator (DS7A, Digitimer Ltd., Hertfordshire, United Kingdom). Single stimuli, square wave pulse of 1 ms duration, were delivered to the muscle while participants maintained a 20% MVC, and the intensity of stimulation was increased until a plateau in twitch amplitude and M-wave occurred. Supramaximal stimulation was achieved by increasing the final stimulator output intensity by a further 30%. The sEMG electrode remained in place as resting twitch peak force was recorded and M-wave peak-to-peak amplitude calculated. Supramaximal stimulation was then applied on top of the MVC procedure to allow calculation of voluntary activation.

#### 2.4.6 Blood sample collection and analysis

Blood samples were collected from an antecubital vein and stored at −80 °C before fatty acid analysis by OmegaQuant Analytics, LLC (Sioux Falls, USA) as described previously [11].

### 2.5 Sample size

The sample size for the main trial was calculated based on the primary outcome of knee extensor maximal strength. Although no minimally clinically important difference (MCID) has been established, estimates suggest a threshold of 4–6% [17]. Assuming an SD of 9% from pilot data in our laboratory, a sample size of 50 participants per group provided 80% power at p < 0.05. The study was not powered to detect subgroup interactions, and the current analyses are, therefore, exploratory.

### 2.6 Statistical analysis

Data were analysed using R (v2025.05.1). Knee extensor maximal torque, grip strength, and muscle thickness were compared using mixed-design ANOVA (afex package), with Time as a within-subject factor and Group and Sex/Age/BMI as between-subject factors. Estimated marginal means were calculated (emmeans package), and post-hoc pairwise comparisons were adjusted using the Bonferroni method. Statistical significance was accepted at p < 0.05.

## 3 Results

### 3.1 Participant characteristics

Ninety-four participants with complete data were included in this analysis. Baseline characteristics, stratified by Group and by Sex/Age/BMI, are presented in Table 1. In the krill oil group, the omega-3 index increased from 5.8% (1.2) to 9.0% (2.1) in men and from 7.1% (1.9) to 10.8% (1.8) in women. No change was observed in the control groups, and no sex differences were detected.

**Table 1:** Participant baseline characteristics stratified by Group and Sex,Age. BMI.

	Control: Female (n = 27)	Krill: Female (n = 26)	Control: Male (n = 18)	Krill: Male (n = 23)
Age (years)	70.6 (5.3)	70.6 (4.0)	71.3 (5.2)	72.0 (5.7)
Height (cm)	158 (6)	163 (6)	174 (6)	176 (5)
Body Mass (kg)	63.7 (11.1)	65.7 (7.6)	82.2 (10.8)	80.1 (7.5)
BMI (kg/m^2^)	25.41 (4.24)	24.71 (2.69)	27.23 (3.43)	25.93 (2.57)
Systolic Blood Pressure (mmHg)	131 (14)	132 (13)	132 (14)	139 (10)
Diastolic Blood Pressure (mmHg)	76 (9)	78 (9)	81 (9)	79 (7)

	Control: Young (n = 27)	Krill: Young (n = 27)	Control: Old (n = 18)	Krill: Old (n = 22)
Age (years)	67.48 (1.70)	67.78 (1.72)	76.06 (4.32)	75.55 (4.00)
Height (cm)	164.41 (10.44)	169.26 (8.06)	164.66 (9.62)	168.98 (9.26)
Body Mass (kg)	72.13 (15.75)	72.53 (9.83)	69.53 (11.77)	72.44 (11.32)
BMI (kg/m^2^)	26.46 (4.19)	25.27 (2.53)	25.65 (3.76)	25.31 (2.91)
Systolic Blood Pressure (mmHg)	127.32 (12.81)	135.14 (12.61)	138.15 (12.56)	135.76 (11.32)
Diastolic Blood Pressure (mmHg)	77.41 (9.29)	79.42 (8.22)	79.94 (9.18)	77.50 (7.39)

	Control: Normal Weight (n = 18)	Krill: Normal Weight (n = 24)	Control: Overweight/Obese (n = 27)	Krill: Overweight/Obese (n = 26)
Age (years)	70.06 (5.17)	70.21 (4.00)	71.48 (5.22)	72.28 (5.49)
Height (cm)	162.67 (10.83)	169.10 (8.35)	165.74 (9.42)	169.16 (8.86)
Body Mass (kg)	59.19 (9.53)	66.44 (7.19)	79.02 (10.91)	78.30 (9.77)
BMI (kg/m^2^)	22.26 (1.68)	23.20 (1.47)	28.72 (2.81)	27.29 (1.94)
Systolic Blood Pressure (mmHg)	127.41 (15.98)	129.65 (11.85)	134.48 (11.32)	140.95 (9.21)
Diastolic Blood Pressure (mmHg)	73.52 (9.97)	77.25 (8.83)	81.69 (7.17)	79.81 (6.70)

Data are mean (SD).

### 3.2 Sex-differences

**Fig. 1:**
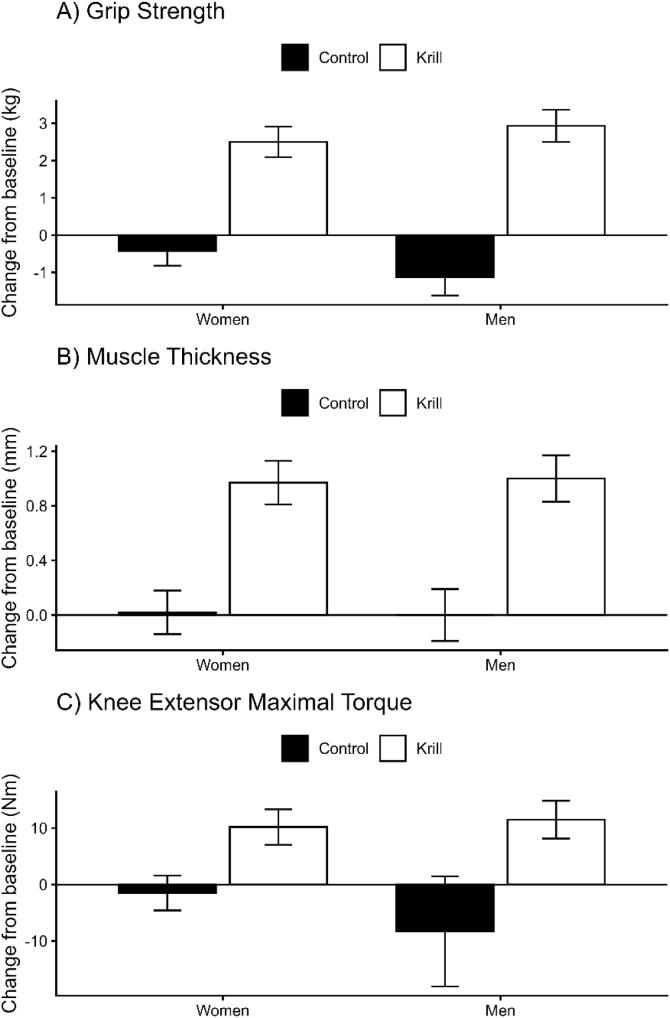
Changes in (A) grip strength, (B) muscle thickness, and (C) knee extensor maximal torque in men and women following intervention with krill oil or control stratified by sex. Values are mean ± SE. Krill subgroups showed significant increases in all measures, whereas control subgroups showed no change (all P < 0.05).

Outcomes are presented in Table 2 with changes in the main outcomes of strength visualised in Fig. 1. Time (p < 0.001) and Group*Time (p < 0.001), but no Group*Sex*Time interaction (p = 0.603) effects were observed for the omega-3 index. Analysis of knee extensor maximal torque data revealed no effect of Time (p = 0.093) or Group*Sex*Time interaction (p = 0.206), but a Group*Time interaction was noted (p < 0.001). The mean difference in change between krill and control group, the krill treatment effect, was 11.70 Nm (95%CI: 3.05–20.35 N m) in females and 20.24 Nm (95%CI: 10.34–30.15 N m) in males. Analysis of grip strength data revealed effects of Time (p < 0.001) and Group*Time (p < 0.001), with no Group*Sex*Time interaction (p = 0.188). The mean difference in change between krill and control group, the krill treatment effect, was 2.93 kg (95%CI: 1.81–4.04 kg) in females and 4.07 kg (95%CI: 2.80–5.35 kg) in males. Analysis of muscle thickness data revealed effects of Time (p < 0.001) and Group*Time (p < 0.001), with no Group*Sex*Time interaction (p = 0.893). The mean difference in change between krill and control group, the krill treatment effect, was 0.95 mm (95%CI: 0.51–1.38) in females and 0.99 mm (95%CI: 0.49–1.48 mm) in males. Analysis of time taken to walk 4 m data found no Time (p = 0.591), Group*Time (p = 0.802) or no Group*Sex*Time interaction (p = 0.982). Analysis of chair rise time data found no Time (p = 0.623), Group*Time (p = 0.419) or no Group*Sex*Time interaction (p = 0.915).

**Table 2:** Muscle strength and size data before and after the intervention period, stratified by Group and Sex.

	Control		Krill		Control		Krill	
	Baseline	6 Months	Baseline	6 Months	Baseline	6 Months	Baseline	6 Months
Omega-3 Index (%)	7.07 (2.12)	6.62 (1.73)	7.12 (1.93)	10.83 (1.78)	6.24 (1.51)	6.37 (1.41)	5.84 (1.20)	9.01 (2.06)
Knee extensor maximal torque (Nm)	91.05 (23.42)	89.56 (21.31)	107.55 (28.30)	117.76 (32.55)	162.12 (36.54)	153.33 (36.93)	153.85 (36.67)	165.30 (34.44)
Grip strength (kg)	24.07 (4.13)	23.65 (4.14)	25.94 (4.32)	28.44 (4.67)	38.69 (7.79)	37.56 (7.46)	39.96 (6.54)	42.89 (7.18)
Muscle thickness (mm)	31.50 (5.62)	31.52 (5.62)	31.22 (5.94)	32.19 (6.34)	38.12 (5.18)	38.12 (5.19)	35.27 (5.71)	36.27 (5.69)
4 m walk time (s)	4.25 (0.39)	4.23 (0.40)	4.24 (0.51)	4.22 (0.51)	4.23 (0.47)	4.24 (0.46)	4.21 (0.63)	4.21 (0.61)
Chair rise time (s)	11.89 (1.26)	11.84 (1.02)	11.70 (1.13)	11.72 (1.13)	11.87 (1.20)	11.83 (1.17)	12.16 (1.29)	12.17 (1.26)
Voluntary activation (%)	76.42 (8.86)	79.02 (7.58)	75.24 (6.43)	78.74 (9.19)	80.54 (6.09)	81.32 (5.06)	76.64 (12.05)	83.84 (10.71)
RTD50 (N ms-1)	157.54 (128.64)	148.44 (102.31)	201.31 (172.87)	217.65 (219.28)	630.06 (585.79)	459.06 (354.21)	396.71 (300.75)	451.00 (269.76)
RTD100 (N ms-1)	108.13 (85.05)	114.29 (80.00)	138.82 (100.32)	155.50 (126.67)	350.98 (310.29)	295.05 (189.20)	235.13 (201.50)	285.09 (156.13)
RTD200 (N ms-1)	104.70 (70.99)	105.33 (62.68)	139.62 (89.56)	142.96 (91.79)	298.65 (213.13)	262.40 (155.39)	229.95 (129.43)	262.13 (118.17)
RTD300 (N ms-1)	103.11 (61.49)	102.64 (58.03)	129.09 (65.65)	137.31 (70.14)	252.07 (152.43)	229.01 (120.27)	212.23 (93.38)	231.37 (94.49)
RMS (uV)	58.55 (36.21)	58.97 (27.35)	65.42 (42.03)	67.93 (43.17)	146.59 (66.89)	128.18 (49.56)	152.34 (82.34)	156.95 (93.78)
M-wave (mV)	19.59 (4.06)	18.35 (3.66)	19.53 (4.80)	20.24 (6.11)	20.94 (6.48)	17.13 (5.15)	17.26 (4.68)	21.06 (4.64)

Data are mean (SD).

Analysis of voluntary activation data found an effect of Time (p < 0.001), with no Group*Time (p = 0.070) or Group*Sex*Time interaction (p = 0.170) effects found. Analysis of RTD50 data found no Time (p = 0.356), Group*Time (p = 0.066) or no Group*Sex*Time interaction (p = 0.094). Analysis of RTD100 data found no Time (p = 0.784), Group*Time (p = 0.061) or no Group*Sex*Time interaction (p = 0.124). Analysis of RTD200 data found no Time (p = 0.998), Group*Time (p = 0.061) or no Group*Sex*Time interaction (p = 0.083). Analysis of RTD300 data found no Time (p = 0.890), Group*Time (p = 0.069) or no Group*Sex*Time interaction (p = 0.229). Analysis of RMS data found no Time (p = 0.505), Group*Time (p = 0.125) or no Group*Sex*Time interaction (p = 0.200). Analysis of Mwave data found no Time (p = 0.804) effect, but Group*Time (p < 0.001) and Group*Sex*Time interaction (p = 0.009) effects were noted. Post-hoc analysis of subgroups found no change over time in females in either the control or krill groups, but found an increase in M-wave in males in the krill group (+3.80 [1.72–5.88] mV, p = 0.016) with a tendency for a decrease in the control group (−3.71 [1.58–6.05] mV, p = 0.059). The mean difference in change between krill and control group, the krill treatment effect, was 1.94 mV (95%CI: −0.80–4.69 mV) in females and 7.61 mV (95%CI: 4.46–10.76 mV) in males.

### 3.3 BMI-differences

**Fig. 2:**
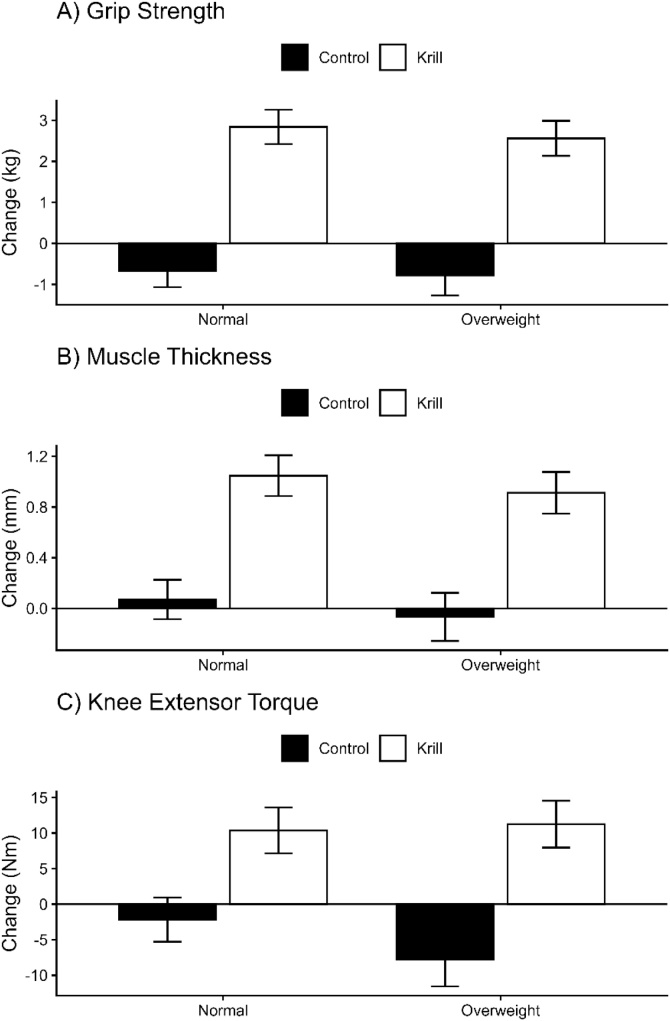
Changes in (A) grip strength, (B) muscle thickness, and (C) knee extensor maximal torque in men and women following intervention with krill oil or control stratified by BMI (normal weight ≤24.9 kg/m^2^ and overweight/obese >25 kg/m^2^). Values are mean ± SE. Krill subgroups showed significant increases in all measures, whereas control subgroups showed no change (all P < 0.05).

Outcomes are presented in Table 3 with changes in the main outcomes of strength visualised in Fig. 2. As Time and Group*Time effects have been presented in the previous section these will not be repeated moving forward. No Group*BMI*Time interaction (p = 0.834) effects were observed for the omega-3 index. No Group*BMI*Time interactions were noted for knee extensor maximal torque (p = 0.340), with the mean difference in change between the krill and control group, the krill treatment effect, being 19.01 Nm (95% CI: 9.14–28.87 N m) in the normal-BMI subgroup and 12.54 Nm (95% CI: 3.76–21.32 N m) in the overweight/obese subgroup. Similarly, no Group*Age*Time interactions were noted for grip strength (p = 0.950), with the krill treatment effect being 3.34 kg (95% CI: 2.06–4.62 kg) in normal-BMI participants and 3.51 kg (95% CI: 2.37–4.64 kg) in overweight/obese participants. Furthermore, no Group*Age*Time interactions were noted for muscle thickness (p = 0.893), with the krill treatment effect being 0.98 mm (95% CI: 0.49–1.47 mm) in the normal-BMI subgroup and 0.98 mm (95% CI: 0.54–1.42 mm) in the overweight/obese subgroup. Finally, no Group*Age*Time interactions were noted for time to walk 4 m (p = 0.893); or chair rise time (p = 0.626).

**Table 3:** Muscle strength and size data before and after the intervention period, stratified by Group and BMI Category.

					Overweight/Obese			
	Control		Krill		Control		Krill	
	Baseline	6 Months	Baseline	6 Months	Baseline	6 Months	Baseline	6 Months
Omega-3 Index (%)	7.39 (2.06)	6.62 (1.43)	6.55 (2.13)	10.79 (1.86)	6.31 (1.74)	6.45 (1.72)	6.49 (1.30)	9.19 (2.05)
Knee extensor maximal torque (Nm)	119.36 (49.59)	111.60 (45.89)	118.04 (27.32)	129.29 (32.72)	119.56 (43.72)	117.38 (40.55)	140.08 (46.83)	150.44 (45.67)
Grip strength (kg)	29.06 (9.26)	28.28 (9.58)	30.52 (7.45)	33.08 (7.99)	30.50 (9.42)	29.83 (8.54)	34.44 (9.87)	37.28 (10.29)
Muscle thickness (mm)	31.32 (5.50)	31.26 (5.53)	29.71 (4.51)	30.62 (4.56)	36.03 (6.19)	36.10 (6.13)	36.39 (5.72)	37.44 (6.03)
4 m walk time (s)	4.15 (0.38)	4.18 (0.38)	4.10 (0.54)	4.14 (0.55)	4.30 (0.43)	4.27 (0.45)	4.35 (0.56)	4.28 (0.57)
Chair rise time (s)	11.40 (1.48)	11.32 (1.14)	11.62 (1.17)	11.66 (1.18)	12.21 (0.91)	12.17 (0.89)	12.19 (1.22)	12.19 (1.19)
Voluntary activation (%)	79.87 (8.56)	81.18 (7.89)	75.66 (8.87)	82.13 (8.09)	76.58 (7.86)	78.97 (6.14)	76.08 (9.93)	79.96 (11.91)
RTD50 (N ms-1)	274.26 (313.72)	217.55 (227.70)	270.21 (181.73)	353.29 (309.04)	394.74 (513.11)	309.44 (308.50)	314.93 (317.47)	302.12 (227.20)
RTD100 (N ms-1)	182.41 (193.07)	153.74 (119.18)	158.53 (71.37)	233.61 (172.69)	220.51 (264.02)	208.50 (181.23)	208.50 (215.09)	199.74 (135.76)
RTD200 (N ms-1)	161.51 (146.90)	142.64 (104.44)	167.34 (73.62)	203.35 (122.02)	196.13 (189.10)	185.17 (148.60)	196.12 (149.15)	194.63 (120.39)
RTD300 (N ms-1)	149.74 (119.52)	137.87 (93.60)	154.92 (55.48)	184.83 (85.95)	171.33 (136.58)	163.40 (116.02)	180.79 (112.72)	178.22 (103.48)
RMS (uV)	105.79 (70.52)	86.58 (38.89)	87.17 (55.30)	98.43 (71.02)	85.75 (63.55)	86.71 (57.92)	124.50 (90.86)	120.55 (94.60)
M-wave (mV)	21.02 (5.97)	18.59 (4.40)	18.70 (4.08)	19.63 (5.38)	19.54 (4.52)	17.38 (4.26)	18.24 (5.54)	21.59 (5.41)

Data are mean (SD).

**Figure 2 here**

Similarly, no Group* BMI *Time interactions were noted for voluntary activation (p = 0.353), RTD50 (p = 0.574), RTD100 (p = 0.102), RTD200 (p = 0.231), RTD300 (p = 0.187), or M-wave (p = 0.328). For M-wave, the krill treatment effect was 3.35 mV (95% CI: 0.16–6.55 mV) in normal-BMI participants and 5.50 mV (95% CI: 2.66–8.34 mV) in overweight/obese participants. Group* BMI *Time interactions were noted for RMS (p = 0.030), although post-hoc tests found no differences in responses between groups (all p > 0.05).

### 3.4 Age-differences

**Fig. 3:**
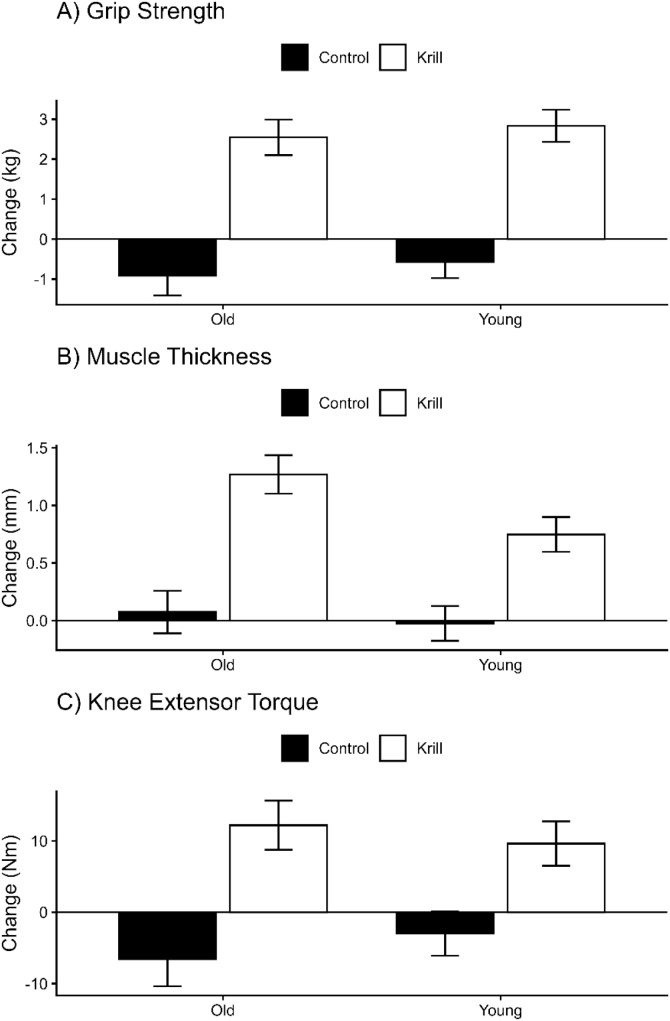
Changes in (A) grip strength, (B) muscle thickness, and (C) knee extensor maximal torque in men and women following intervention with krill oil or control stratified by age (young ≤70 years and old >70 years). Values are mean ± SE. Krill subgroups showed significant increases in all measures, whereas control subgroups showed no change (all P < 0.05).

Outcome are presented in Table 4 with changes in the main outcomes of strength visualised in Fig. 3. As Time and Group*Time effects have been presented in the previous section these will not be repeated moving forward. No Group*Age*Time interaction (p = 0.474) effects were observed for the omega-3 index. Analysis of knee extensor maximal torque revealed no Group*Age*Time interaction (p = 0.367). The mean difference in change between the krill and control group, the krill treatment effect, was 18.77 Nm (95% CI: 8.68–28.85 N m) in older adults and 12.63 Nm (95% CI: 3.99–21.26 N m) in younger adults. Grip strength revealed no Group*Age*Time interaction (p = 0.950). The krill treatment effect was 2.93 kg (95% CI: 1.81–4.04 kg) in older adults and 4.07 kg (95% CI: 2.80–5.35 kg) in younger adults. Muscle thickness revealed no Group*Age*Time interaction (p = 0.202). The krill treatment effect was 1.19 mm (95% CI: 0.70–1.68 mm) in older adults and 0.77 mm (95% CI: 0.35–1.19 mm) in younger adults. Analysis of time to walk 4 m revealed no Group*Age*Time interaction (p = 0.415), and chair rise time also showed no interaction (p = 0.613).

**Table 4:** Muscle strength and size data before and after the intervention period, stratified by Group and Age Category.

	Control		Krill		Control		Krill	
	Baseline	6 Months	Baseline	6 Months	Baseline	6 Months	Baseline	6 Months
Omega-3 Index (%)	6.63 (1.80)	6.57 (1.42)	6.18 (1.70)	9.75 (1.96)	6.90 (2.15)	6.45 (1.86)	6.93 (1.73)	10.25 (2.28)
Knee extensor maximal torque (Nm)	119.92 (42.06)	116.94 (42.20)	135.04 (39.84)	144.68 (40.90)	118.81 (51.74)	112.26 (43.64)	122.21 (39.34)	134.43 (41.05)
Grip strength (kg)	30.54 (9.94)	29.96 (9.43)	33.35 (9.18)	36.19 (10.09)	29.00 (8.37)	28.08 (8.16)	31.50 (8.66)	34.05 (8.52)
Muscle thickness (mm)	34.60 (6.69)	34.58 (6.78)	33.32 (6.05)	34.07 (6.25)	33.47 (5.80)	33.54 (5.66)	32.88 (6.34)	34.15 (6.56)
4 m walk time (s)	4.24 (0.49)	4.23 (0.50)	4.14 (0.51)	4.14 (0.56)	4.23 (0.29)	4.23 (0.29)	4.34 (0.61)	4.30 (0.55)
Chair rise time (s)	11.78 (1.37)	11.70 (1.13)	11.62 (1.30)	11.64 (1.27)	12.04 (0.98)	12.03 (0.97)	12.27 (1.03)	12.28 (1.03)
Voluntary activation (%)	76.58 (8.22)	80.83 (6.45)	78.24 (10.61)	84.17 (9.46)	79.10 (8.17)	78.81 (7.24)	73.27 (6.97)	77.61 (9.91)
RTD50 (N ms-1)	326.99 (452.55)	307.66 (325.47)	285.03 (273.19)	412.47 (318.25)	375.89 (442.22)	220.22 (189.49)	302.84 (244.83)	222.51 (137.48)
RTD100 (N ms-1)	199.98 (249.38)	194.45 (167.07)	196.10 (179.82)	261.35 (178.37)	213.21 (222.68)	174.81 (152.99)	169.21 (139.48)	161.07 (95.92)
RTD200 (N ms-1)	184.28 (182.36)	177.17 (143.50)	185.77 (126.20)	232.01 (136.28)	179.28 (161.72)	154.64 (118.36)	177.42 (109.93)	158.27 (82.36)
RTD300 (N ms-1)	169.05 (140.12)	161.57 (115.11)	170.21 (91.37)	203.35 (106.52)	153.16 (113.69)	140.61 (96.02)	165.55 (89.07)	154.60 (70.24)
RMS (uV)	91.79 (61.73)	89.27 (54.09)	115.39 (86.92)	120.88 (98.28)	96.72 (74.57)	82.73 (46.26)	94.97 (63.27)	96.02 (61.01)
M-wave (mV)	19.80 (4.85)	17.54 (4.59)	19.13 (4.83)	21.99 (5.22)	20.62 (5.65)	18.35 (3.92)	17.65 (4.83)	18.95 (5.31)

Data are mean (SD).

Similarly, no Group*Age*Time interactions were noted for voluntary activation (p = 0.444), RTD50 (p = 0.535), RTD100 (p = 0.510), RTD200 (p = 0.196), RTD300 (p = 0.154), RM (p = 0.669) or M-wave (p = 0.485). For M-wave, the krill treatment effect was 3.57 mV (95% CI: 0.29–6.86 mV) in older adults and 5.12 mV (95% CI: 2.31–7.93 mV) in younger adults.

## 4 Discussion

This double-blind randomised controlled trial demonstrated that six months of krill oil supplementation (4 g/day) increased muscle strength and size in older adults, with comparable effects regardless of sex, age or BMI. This finding is important from a public health perspective as it suggests that krill oil supplementation represents a viable strategy to support muscle health in older age, regardless of sex, age or BMI. Interestingly the effects of krill oil on the M-wave were greater in men than in women, indicating that the mechanisms through which krill oil influences muscle strength may differ by sex.

Early epidemiological data suggested possible sex-specific associations between fatty fish intake and muscle strength. In one cohort, each additional weekly portion of fatty fish was associated with a 1.8% higher grip strength in women but only a 1.0% higher strength in men [5]. However, these cross-sectional data cannot establish causality. Beyond the nutritional composition of fish, its consumption may also reflect specific dietary patterns and lifestyle factors that cannot be fully adjusted for in observational studies [16,17]. Interventional studies without concomitant resistance training have not previously compared responses between men and women [18]. In trials combining LCn-3 PUFA with resistance training, sex differences have been observed, with supplementation augmenting strength gains in women but not men [12].

It has been proposed that these sex differences may be attributed to differences in the enrichment of LCn-3 PUFA into cell membranes, as at the same dose of LCn-3 PUFA supplementation, a greater EPA and total LC n–3 PUFA enrichment in plasma phosphatidylcholine is seen in women after fish-oil supplementation [19], although such differences were not seen by Da Boit et al. [12]. In the current study, muscle strength and size increases were similar in men and women in the absence of resistance exercise, alongside similar omega-3 index increases between sexes. This would indicate, therefore, that LCn-3 PUFA, in the form of krill oil, has sex-independent benefits for muscle strength and size in older adults. Although women cross the disability threshold earlier, due to their lower starting strength and greater longevity [20], sarcopenia affects both men and women [1]. Interventions that are effective irrespective of sex, therefore, have greater public health utility. Interestingly, sex-dependent changes in the Mwave were observed in response to krill oil, with greater effects in men compared to women. This indicates that although strength increases were comparable between men and women, the mechanisms underlying these effects may differ and further work should examine this.

Prior to the current study there had been little investigation of whether the effects of LCn-3 PUFA differed according to age or BMI. This is important as it is known that overweight/obesity can influence the effects of age on muscle [21], and that even in older adults advancing age is associated with a more rapid decline in neuromuscular capacity [14]. The current data found that both from a strength and size, and also a mechanistic point of view, krill oil results in comparable increases in size and strength regardless of age or BMI, in healthy older adults. This is important as it reinforces the generalizability of the intervention across a heterogeneous older adult population. Despite the improvements in muscle strength and vastus lateralis thickness observed in the krill oil group, these changes did not translate into measurable improvements in physical function, as assessed by 4-m walk time and chair-rise performance. This likely reflects characteristics of our study population, as participants were generally healthy, community-dwelling older adults with relatively high baseline functional ability, which may have limited the scope for further improvement via ceiling effects in these performance tests [22]. Simple timed measures such as short-distance gait speed and repeated chair-stands show low sensitivity to modest physiological changes, particularly in higher-functioning cohorts and in the absence of a concurrent, task-specific functional training stimulus. The lack of change in these outcomes is, therefore, consistent with reports that gains in muscle mass or strength do not invariably result in detectable improvements in gait or chair-rise performance in well-functioning older adults, especially when interventions do not directly target functional tasks [23]. Across the neuromuscular measures assessed including voluntary activation, rate of torque development (RTD50/100/200/300), and RMS amplitude changes over the intervention period were small and showed considerable inter-individual variability. These outcomes are known to exhibit greater measurement noise compared to strength or muscle thickness, limiting power to detect differences in these outcomes Accordingly, the absence of significant subgroup effects likely reflects a combination of limited statistical power, higher inherent variability in neuromuscular assessments, and the exploratory nature of these analyses. This context is important for interpreting the predominantly null findings, and future work with larger samples is required to more definitively determine whether neuromuscular responses to LCn-3 PUFA differ by sex, age, or BMI. The current study is not without limitations and in particular it is prudent to not again that the study was not powered to detect subgroup interaction effects and so these findings must be considered exploratory in nature, and require confirmation in further studies.

In summary, this double-blind randomised controlled trial demonstrated that six months of krill oil supplementation produced comparable muscle strength and size increases in healthy older adults, regardless of their sex, age or BMI, although the mechanisms of action may differ by sex. These findings suggest that krill oil may effectively counter age-related declines in muscle mass and function, regardless of sex, age or BMI in healthy older adults.

## Funding

SA was funded by a studentship from the Government of Saudi Arabia.

## Declaration of competing interest

The authors declare no conflicts of interest.
